# Microbiome evolution during host aging

**DOI:** 10.1371/journal.ppat.1007727

**Published:** 2019-07-25

**Authors:** Francisco Daniel Davila Aleman, Dario Riccardo Valenzano

**Affiliations:** Max Planck Institute for Biology of Ageing, Cologne, Germany; Tufts Univ School of Medicine, UNITED STATES

## Host–microbiota interactions

Commensal microbes and their multicellular eukaryotic hosts constitute a highly integrated system—termed the holobiont [1]—which undergoes dynamic changes through time as it integrates and responds to signals from the environment.

Dwelling at the interface between host epithelia and the external environment, commensal microbes actively modulate development, nutrient absorption, and disease onset in the host. Host metabolism is significantly modulated by commensal microbes, and the gut microbial composition significantly affects blood metabolite composition [2].

Microbial communities differ among epithelia, reaching the highest complexity and taxonomic diversity in the oral cavity and in the gastrointestinal tract [3, 4]. Environmental factors, such as diet, drug use, and social environment, shape the composition of epithelia-associated microbiota [5–7], and environmental heterogeneity—rather than host genetics—can explain much of the interindividual differences in microbiota composition in humans [8]. The assembly of specific host-associated communities, however, is also dictated by the host cell composition and activity, by the molecular components of the mucus layer, by the gut peristaltic contractility [9], and by epithelial integrity [10]. In primates, recent evidence supports that host phylogenetic relatedness and gut physiology are overall better predictors of microbiota composition than diet [11]. Together, the microbiota is a dynamic community, subject to changes in conjunction with host evolution and through the lifetime of individual hosts.

## Microbiota changes through time

Just as the composition of the microbiota varies within and between tissues [12], microbial consortia do also vary through time within individual tissues. Microbial composition in the gut of newborns is dramatically shaped by diet and varies depending on whether the infant is fed with maternal milk [13] or formula [14]. Drug administration and antibiotic use importantly shape the host gut microbiota, leading to significant community shifts and increased abundance of otherwise rare microbial taxa [15]. Although individual gut microbiota are largely unstable in the first years of life, they become more stable during adulthood [13, 16] and undergo dramatic changes in richness and composition upon onset of disease and frailty [17, 18]. The onset of specific diseases, such as cancer, obesity, diabetes, or inflammatory bowel disease (IBD), is associated with specific microbial signatures [19, 20]. Studies in humans and laboratory model organisms, such as flies, fish, and mice, have additionally shown that the composition of the gut microbiota dramatically changes during aging and is associated with host health and life span [17, 21–24]. In mice, e.g., lipopolysaccharide (LPS) from gut microbiota can accelerate age-dependent inflammation (“inflammaging”) [25], and mice lacking Toll-Like receptor 4 (TLR4), which is the LPS receptor, are protected from age-dependent inflammation [26], showing that a microbial-specific substrate induces aging-specific phenotypes. Inflammaging can be further exacerbated in germ-free mice by gut microbiota transfers from aged donor mice [27], showing a direct causal relation between age-specific microbial communities and host aging.

Using deep learning to analyze human microbiome data helped build a human microbiome aging clock, which predicts host age with an accuracy of about 4 years [28]. While during adulthood microbial composition contributes to cellular and tissue homeostasis [29, 30], age-dependent changes in the microbial composition may contribute to increasing frailty and disease onset in later life. The causes leading to the changes in microbiota composition and function during host aging are still poorly understood and possibly include direct or indirect microbial selection by the host and microbe–microbe interactions, as well as microbial evolution.

## Host aging induces shifts to the microbial niche

**Fig 1:**
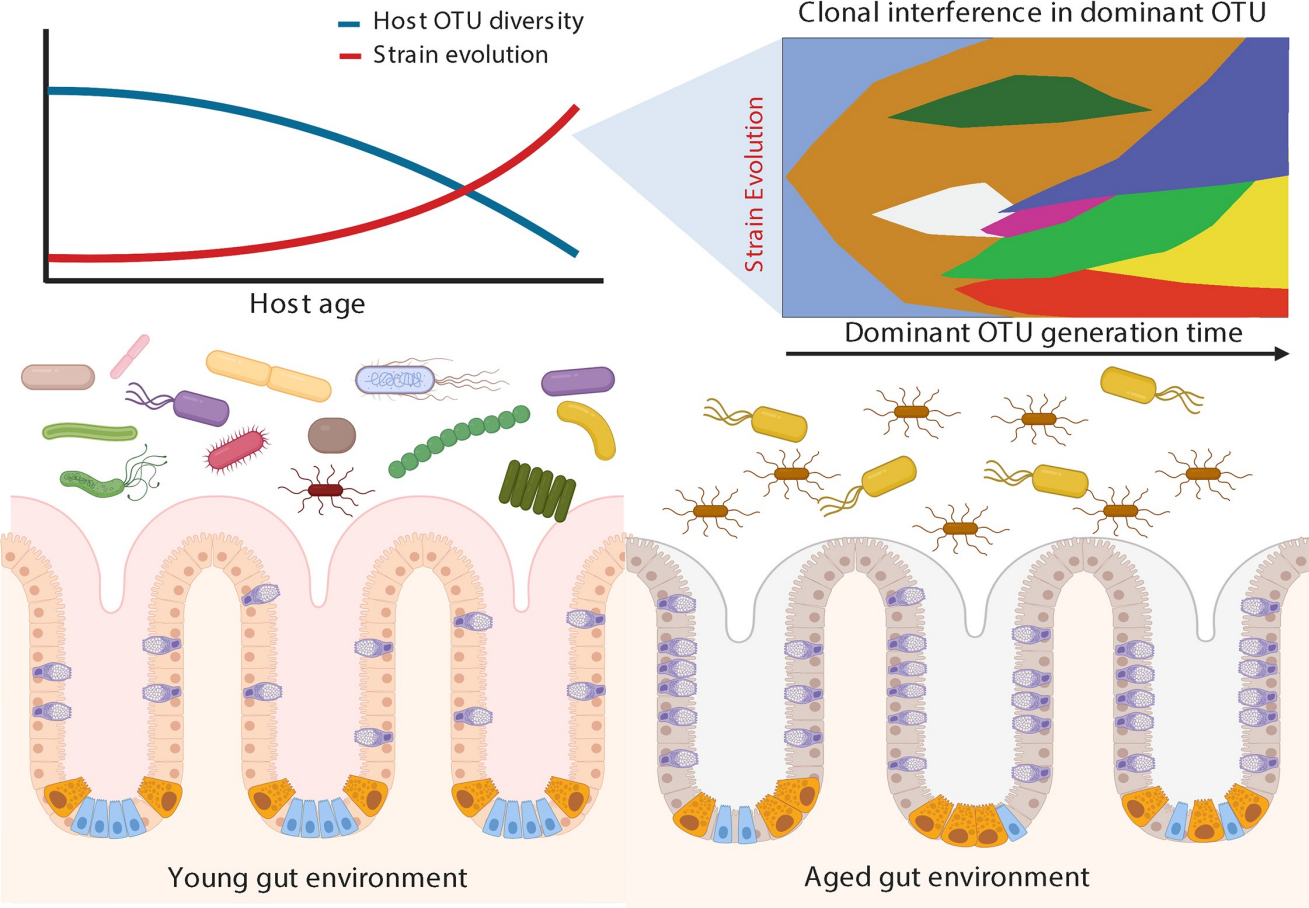
The gut microbiota undergoes dynamic changes during host aging. Changes in host intestinal cell composition and architecture occurring during aging are matched by a decrease in the microbiota taxonomic diversity. Age-related decrease in taxonomic diversity in the commensal community leads to larger population size for a few age-associated microbial species, increasing the chances for the evolution of novel potentially pathogenic microbial strains. OTU, Operational Taxonomic Unit. This figure was generated wtih Biorender.

As they age, organisms accumulate molecular damage (e.g., in DNA and proteins) [31, 32], dysfunctional organelles [33], and senescent cells [34] and undergo compositional changes in the extracellular compartment [35, 36]. Together, these molecular and functional changes lead to organ and systemic decline, which ultimately results in death. Constantly exposed to a changing environment, the microbiota dynamically respond by altering both metabolic function and individual bacterial species composition. The immune system of the host plays a key role in shaping commensal microbial communities by selectively eliminating pathogens and allowing commensals to thrive. During aging, progressive or sudden immune dysfunction and generalized inflammation lead to improper surveillance at the interface between the host and the microbiota, which can result in dysbiosis—an imbalance in bacterial community composition [37]. In humans, young-associated microbiota are enriched with bacterial taxa shown to have immune-modulatory functions, such as Clostridiales and *Bifidobacterium*, whereas old-associated bacterial communities are enriched with pathobionts—e.g., Enterobacteriaceae—and, overall, have a higher representation of Proteobacteria [23, 38]. Here, we argue that the shifting host environment occurring in the time scale of host life is compatible with inter- and intraspecies microbial competition and with the evolution of novel bacterial strains that could become overrepresented in older hosts, leading to emergence of pathogenic strains that may contribute to age-dependent host decline (Fig 1). Age-dependent immune decline could therefore enable the evolution of bacterial strains responsible for elderly-specific bacterial infections.

## Evolution in commensal bacteria

Studies in both germ-free and conventionally raised laboratory mice, which carry a taxonomically complex microbial community, have shown that bacteria in the gut acquire several advantageous mutations, de facto evolving [39] both in short (months) and long (years) time scales [40]. Changes in mutation rates, emergence of novel individual gene variants, and widespread horizontal gene transfer are essential for microbial adaptations, enabling evolution of drug (e.g., antibiotics) resistance and dynamic response to dietary changes [41]. Experiments in mice colonized with *Escherichia coli* have shown clonal interference and parallel phenotypic evolution in the gut, occurring from the emergence of several adaptive genetic variants that reach intermediate frequencies, rather than reaching fixation (i.e., maximum frequency), within individual bacterial species. The coexistence of several strains carrying adaptive variants, each at intermediate frequencies (also known as soft sweeps), sustains genetic diversity within bacterial species of the microbiota [39]. Microbial adaptation in the gut in response to specific selective regimes, such as antibiotics, shows convergent evolution at the gene-variant and functional level [40]. In humans, gut commensal microbes undergo local adaptation and bona fide evolution of new strains via nucleotide substitution and recombination in short time scales, whereas ecological dynamics—consisting in species replacement—are the dominant mechanisms over longer time scales, e.g., decades [42]. Multiple independent lineages of *Bacteroides fragilis*, each carrying independent small and large-scale genetic variants, are detected in healthy humans [43], showing unique within-individual evolutionary trajectories of commensal microbes.

## Microbial evolution during host aging

Although we are now starting to understand how bacterial taxonomic composition and diversity change during different stages of individual life—including during the aging process—we still know very little about whether bacterial evolution plays an important functional role that can impact host phenotypes and ultimately fitness. We have limited understanding on whether the changes in taxonomic composition of host microbiota occurring through host life in healthy individuals are uniquely due to ecological processes (e.g., species replacement) or whether they are, at least in part, due to bacterial evolution. We still do not know whether bacterial evolution participates in the changes in microbiota composition that occur upon the onset of aging-associated diseases. If bacterial evolution does affect host phenotypes, e.g., by enabling specific bacterial taxa to escape immune surveillance or by modulating antibacterial responses, do bacterial strains keep evolving across multiple hosts, or is bacterial evolution always local and ends with host death? Studying bacterial evolution within individual host-associated microbiota and throughout the time scale of individual host life presents several technical challenges, but it is becoming ever more accessible due to the increased throughput, accuracy, and resolution reached in genome sequencing and analysis [44]. Furthermore, the integration of multi-omics approaches, which combine genomics and metabolomics of gut microbiota, enables accurate identification and phenotyping of commensal bacteria associated with a broad set of host physiological states [45]. Experimental work done in nematode worms has shown that the resident microbiota can foster mutualism (i.e., reciprocal benefit) with the host by evolving novel defense mechanisms that serve the purpose of excluding potential pathogens [46]. However, it is not clear whether evolution of novel microbe-mediated microbial exclusion also contributes to the community shifts in microbial composition observed during host aging. Screening a library of mutant *E*. *coli* for effects on nematode worm survival and aging has shown that a set of mutant strains beneficially affect host mitochondrial unfolded protein responses via the secretion of the polysaccharide colanic acid, resulting in increased worm life span [47]. Similar to the way experimenters test sets of different mutants under laboratory conditions, ongoing microbial evolution in healthy hosts leads to the continuous emergence and extinction of bacterial strains that may have either anti- or pro-longevity effects. However, while experimental nematodes are generally fed a specific *E*. *coli* strain (OP50) [48], complex microbiota likely mask the impact on host fitness of individual bacterial strains emerging within specific bacterial species. For a bacterial strain to impact host physiology and fitness, it is necessary to first succeed among competing strains, including the ancestral strain, and then become a functionally relevant member of the microbiota. It is therefore likely that novel strains may have higher chances to succeed in simple microbial communities, characterized by lower taxonomic complexity. Since during aging and frailty the overall microbial taxonomic diversity declines, it may indeed become more likely for new strains within dominant taxa to sweep to high frequency and affect the host.

## Aging modulation via young-associated gut microbes

Whether microbial evolution in the time scale of individual life affects homeostatic processes within the host is still an open question. Experimental research provides us with important insights into how manipulating the microbiota can significantly affect host health. Combining a specific diet with genetically engineered *E*. *coli* that bind colorectal cancer cells, it was recently possible to achieve cancer prevention and regression in a mouse model of colorectal cancer [49]. Genetically engineering microbes could indeed be a therapeutic strategy to compensate for genetic and metabolic deficiencies and potentially improve host health [50]. Commensal microbes have been proposed as a therapeutic target for cancer immunotherapy [51] and could be even targeted for interventions aimed at counteracting the metabolic dysfunctions occurring during aging. Recent work in model organisms indicates that host-associated bacteria have the potential to beneficially modulate host health, aging, and life span [18, 23, 52]. Commensal microbes do play a key role in several phenotypic and metabolic changes associated with aging. For instance, the age-dependent onset of insulin resistance has recently been associated with the action of commensal microbes with the host immune system [18]. Work with the naturally short-lived African turquoise killifish (*Nothobranchius furzeri*) [53, 54] has shown that acute transfer of gut microbes from young donor individuals to middle-age recipients, after antibiotic treatment, is sufficient to significantly extend life span and delay behavioral aging [23]. Metabolic and cellular changes occurring during aging, coupled with immune senescence and inflammaging [55], generate new metabolic and cellular niches, which could lead to competition and potentially create novel selective constraints for the evolution of new strains within dominant bacterial taxa.

## Conclusion

The interactions between the host and its commensal microbes reach homeostatic balance during youth and adulthood, resisting insults from several external factors, including pathogens. Perturbations to this homeostatic balance can derive from changes in the environment, in diet, and from exposure to drugs such as antibiotics. However, challenges to the host–microbiota balance can also derive from intrinsic factors within the host, i.e., from the vast constellation of alterations that occur during the aging process, including cellular senescence, inflammation, and cancer. On the other hand, microbe–microbe interactions within the host could in principle also lead to host–microbiota disbalance, which could in turn contribute to host aging. Whether the microbiota adapt to the physiological changes occurring during host aging, or whether they actively participate to host dysfunction, remains an important open question. Understanding host–microbiota dynamics during host aging will critically inform future therapeutic interventions. If the microbiota exacerbate the cellular, tissue, and systemic changes that occur during host aging, then targeting the microbiota could, in theory, help therapeutically relieve some of the aging-related pathologies but would, in principle, not impact systemic aging. On the other hand, if the microbiota causally participate in triggering host aging, then interventions that target the microbiota could result in systemic, preventative, and bona fide anti-aging interventions.
